# Supplementary material for: Unsupervised Exercise Training Was Not Found to Improve the Metabolic Health or Phenotype over a 6-Month Dietary Intervention: A Randomised Controlled Trial with an Embedded Economic Analysis
Source: Int J Environ Res Public Health. 2021 Jul 28;18(15):8004. doi: 10.3390/ijerph18158004 (PMC8345544; doi:10.3390/ijerph18158004)
Supplement: Supplementary file 1 [file ijerph-18-08004-s001.zip › ijerph-1276112-supplementary.pdf]

**Table S1.** Clinical outcome parameters in each group (mean ± SD) at each time point (column 2-3-4) and the corresponding p-value of the change over time (column 5-6-7).

| Clinical outcome parameters | Baseline | Month 3 | Month 6 | Change (overtime) month 3 vs. Baseline | Month 6 vs. Baseline | Month 3 vs. Month 6 |
|---|---|---|---|---|---|---|
| **BMI (kg/m²)** | | | | | | |
| UC group | 32.27 ± 3.50 | 30.27 ± 3.97 | 29.91 ± 4.44 | <0.01 | <0.01 | 0.35 |
| I group | 32.98 ± 3.60 | 31.01 ± 3.48 | 31.06 ±3.79 | <0.01 | <0.01 | 0.59 |
| **IHL content (/water)** | | | | | | |
| UC group | 4.37 ± 7.49 | 1.79 ± 2.93 | 1.87 ± 2.74 | 0.03 | 0.04 | 0.7 |
| I group | 7.08 ± 8.95 | 4.66 ± 8.01 | 3.61 ± 3.63 | 0.06 | 0.05 | 0.73 |
| **IMCL (/creatine)** | | | | | | |
| UC group | 7.37 ± 3.82 | 7.31 ± 4.36 | 7.04 ± 4.53 | 0.93 | 0.67 | 0.67 |
| I group | 8.10 ± 5.99 | 7.67 ± 6.99 | 8.57 ±3 7.23 | 0.78 | 0.75 | 0.25 |
| **VAT (cm²)** | | | | | | |
| UC group | 118.78 ± 48.18 | 96.83± 44.77 | 98.91 ± 48.05 | <0.01 | <0.01 | 0.78 |
| I group | 115.27 ± 44.89 | 99.63 ± 38.50 | 99.21 ± 47.78 | <0.01 | <0.01 | 0.36 |
| **SAT (cm²)** | | | | | | |
| UC group | 495.11 ± 113.86 | 439.75 ± 130.13 | 438.65 ± 136.52 | <0.01 | <0.01 | 0.51 |
| I group | 490.87 ± 65.62 | 445.33 ± 91.58 | 452.11 ± 99.16 | <0.01 | <0.01 | 0.18 |
| **PCF (cm³)** | | | | | | |
| UC group | 159.47 ± 55.58 | 148.28 ± 48.49 | 153.65 ± 56.57 | <0.01 | 0.07 | 0.15 |
| I group | 150.81 ± 46.94 | 140.59 ± 43.09 | 150.59 ± 55.72 | 0.17 | 0.83 | 0.39 |
| **ECF (cm³)** | | | | | | |
| UC group | 53.73± 30.32 | 44.00 ± 25.25 | 43.16 ± 26.30 | <0.01 | <0.01 | 0.50 |
| I group | 65.36 ± 54.65 | 49.76 ± 45.00 | 49.64 ± 45.89 | <0.01 | <0.01 | 0.20 |
| **Body Fat (%)** | | | | | | |
| UC group | 39.16 ± 4.28 | 37.52 ± 5.71 | 37.61 ± 5.54 | 0.01 | 0.15 | 0.64 |
| I group | 39.00 ± 3.76 | 37.23 ± 4.71 | 37.95 ± 5.49 | <0.01 | 0.14 | 0.31 |
| **Fat free mass (kg)** | | | | | | |
| UC group | 54.78 ± 5.31 | 52.92 ± 5.23 | 51.93 ± 5.54 | <0.01 | <0.01 | 0.34 |
| I group | 54.11 ± 6.26 | 52.45 ± 6.28 | 51.46 ± 6.39 | <0.01 | <0.01 | 0.14 |
| **Waist circumference (cm)** | | | | | | |
| UC group | 100.09 ± 10.13 | 95.23 ± 8.80 | 91.43 ± 9.96 | <0.01 | <0.01 | <0.01 |
| I group | 101.28 ± 8.35 | 96.73 ± 9.34 | 94.88 ± 9.07 | <0.01 | <0.01 | 0.09 |
| **triglycerides (mg/dL)** | | | | | | |
| UC group | 105.22 ± 69.19 | - | 84.14 ± 46.88 | - | 0.05 | - |
| I group | 99.13 ± 43.19 | - | 87.32 ± 41.84 | - | 0.03 | - |
| **SBP (mmHg)** | | | | | | |

| | | | | | | |
|---|---|---|---|---|---|---|
| UC group | 121.81 ± 14.78 | 120.86 ± 14.10 | 118.05 ± 8.37 | 0.68 | 0.15 | 0.82 |
| I group | 120.00 ± 10.34 | 118.20 ± 8.20 | 118.58 ± 8.79 | 0.25 | 0.62 | 0.86 |
| **DBP (mmHg)** | | | | | | |
| UC group | 72.48 ± 9.94 | 74.43 ± 8.55 | 72.86 ± 6.30 | 0.50 | 0.71 | 0.52 |
| I group | 73.93 ± 9.18 | 74.40 ± 6.05 | 75.58 ± 6.01 | 0.93 | 0.32 | 0.82 |
| **HDL-C (mg/dL)** | | | | | | |
| UC group | 57.19 ± 10.72 | - | 57.24 ± 13.35 | - | 0.56 | - |
| I group | 58.63 ± 11.25 | - | 58.78 ± 10.26 | - | 0.93 | - |
| **Fast. glucose (mg/dL)** | | | | | | |
| UC group | 84.56 ± 7.23 | - | 83.25 ± 8.90 | - | 0.47 | - |
| I group | 86.30 ± 8.57 | - | 86.50 ± 15.93 | - | 0.85 | - |

*Footnote: Pairwise differences between time points were tested using a Tukey correction for multiple comparisons with significance level of 0.05.*
